# Cutaneous Limb Metastasis of Colorectal Cancer Misdiagnosed as Zoster Infection

**DOI:** 10.7759/cureus.36621

**Published:** 2023-03-24

**Authors:** Maram Albandak, Miral Albandak, Jasmin Abdallah, Mohammed Qawasmeh

**Affiliations:** 1 Internal Medicine, Al-Quds University, Jerusalem, PSE; 2 Pharmacy, Birzeit University Faculty of Pharmacy Nursing and Health Professions, Birzeit, PSE

**Keywords:** metastatic skin cancer, adenocarcinoma of colon, skin lesions, colorectal cancer, cutaneous metastasis

## Abstract

Cutaneous metastasis is a rare manifestation of internal malignancies. It usually occurs with the later progression of the disease and is associated with a poor prognosis. Common culprits of skin metastasis include lung cancer, melanoma, and colorectal cancer in men and breast cancer, colorectal cancer, and melanoma in women. Given these points, there is a low rate of cutaneous metastasis of colorectal cancer. When present, the most common sites include the abdominal wall and, less frequently, the face and the scalp. Rarely there is cutaneous metastasis to the upper extremity. Herein, we report the case of a female patient in her 50s who presented with a maculopapular rash of the right upper limb four years after her initial diagnosis of colonic adenocarcinoma. However, because of this rare manifestation, she was initially misdiagnosed with more common causes of a maculopapular rash. After a period of no improvement with preliminary treatment, a biopsy with immunohistochemical staining was undertaken, and the specimen stained positive for CK20 and CDX2, confirming metastatic colorectal malignancy. Skin lesions that are not responding to conventional therapy and those which have bizarre presentations can be a harbinger of internal malignancy and should be considered in the differential.

## Introduction

Colorectal cancer (CRC) is one of the most common forms of cancer around the world and it is the second most common cancer in the West Bank and Jerusalem, with an incidence rate of 13.6 per 100,000 [1,2]. The most common sites of metastasis of colorectal cancer are the liver, lung, regional lymph nodes, central nervous system, and peritoneum. Cutaneous metastases are usually secondary to cancers in the lung and breast and rarely occur secondary to colorectal cancer, occurring in only 4.2% of the cases, and are mostly associated with a poor prognosis [1,3,4]. Cutaneous metastases usually present late in the disease course after the disease has widely spread in the body; however, it is the first sign of internal disease in 16-21% of the cases [1]. We herein present an unusual case of skin metastasis localized to the arm, which occurred during the active treatment of metastatic colorectal adenocarcinoma.

## Case presentation

We present a female patient in her 50s who was diagnosed with stage 4 metastatic colorectal adenocarcinoma in 2017 with right axillary and retroperitoneal nodal involvement. The patient had presented several times prior with severe abdominal pain, worsening constipation, and significant weight loss. Colonoscopy with biopsy and axillary lymph node biopsy confirmed metastatic colorectal adenocarcinoma positive for RAS and BRAF wild type. The patient was started on different chemotherapy regimens including leucovorin calcium (folinic acid), fluorouracil, and oxaliplatin (FOLFOX regimen) and cetuximab, followed by capecitabine and cetuximab with good response, and was then maintained on a single agent cetuximab. Her disease remained stable until 2020, in which an increase in the size of the axillary and pectoral lymph nodes prompted us to switch the chemotherapy regimen back to capecitabine and cetuximab with good response, then to a single agent cetuximab afterwards.

**Figure 1:**
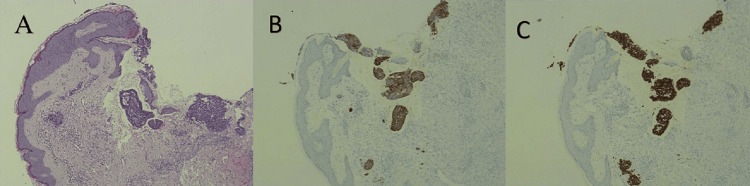
Metastatic colorectal neoplasm in the skin (A-C) A: Hematoxylin and eosin (H&E): Metastatic groups of neoplastic cells showing small glandular and cribriform patterns. B: CK20 shows strong cytoplasmic and membranous positivity in the neoplastic cells. C: The neoplastic cells show positive nuclear staining with CDX2.

Her disease remained stable, until a year later in December 2021, when the patient presented with a right upper limb maculopapular rash associated with swelling and pain. The lesion was initially thought to be an allergic reaction and was treated as such. With no response on anti-allergic medication, the patient was referred to a dermatologist who clinically diagnosed the lesion as herpes zoster and treated the patient with acyclovir. However, no improvement was noted with antiviral treatment, therefore a skin punch biopsy was done shortly after, which confirmed metastatic stage 4 moderately differentiated skin carcinoma of colorectal origin, as shown in Figure 1. No other metastases to other organs were documented. HIV and a prior zoster infection have been ruled out as well. Immunohistochemical staining was positive for cytokeratin 20 (CK20), CDX2 and negative for CK 7 stain, confirming the colorectal origin (Figure 1).

**Figure 2:**
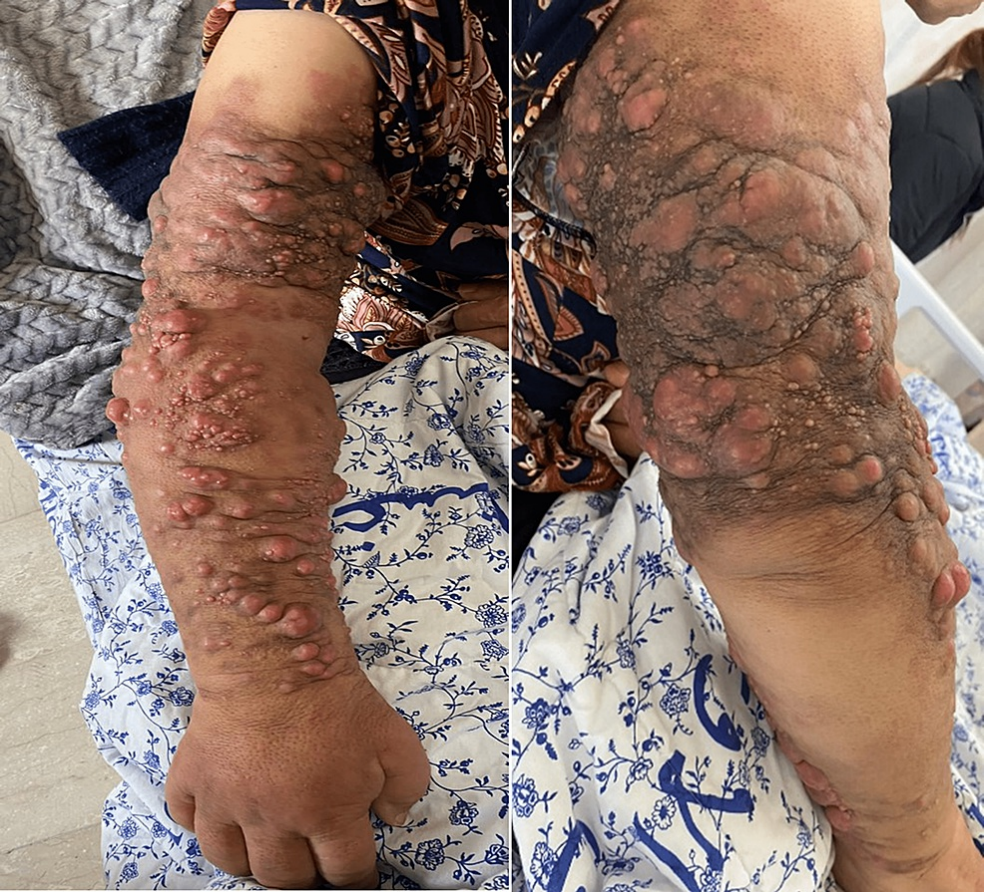
Swollen right extremity (front and back) Multiple violaceous to flesh-colored nodular lesions with associated hyperpigmentation are visible.

The patient was started on multiple different chemotherapy regimens for the skin metastasis; including four cycles of leucovorin calcium (folinic acid), fluorouracil, and irinotecan hydrochloride (FOLFIRI regimen) and bevacizumab, followed by three cycles of FOLFOX and cetuximab due to disease progression on previous line, and again six cycles of FOLFIRI and bevacizumab as a rechallenge. However, her skin lesions continued to progress, from initially multiple skin erythematous lesions associated with diffuse mild edema, to ultimately diffuse skin hyperpigmentation and multiple flesh-colored skin nodules, associated with severe swelling and burning sensation (Figure 2). Eventually, the patient was switched to 10 rounds of palliative radiation therapy.

## Discussion

**Figure 3:**
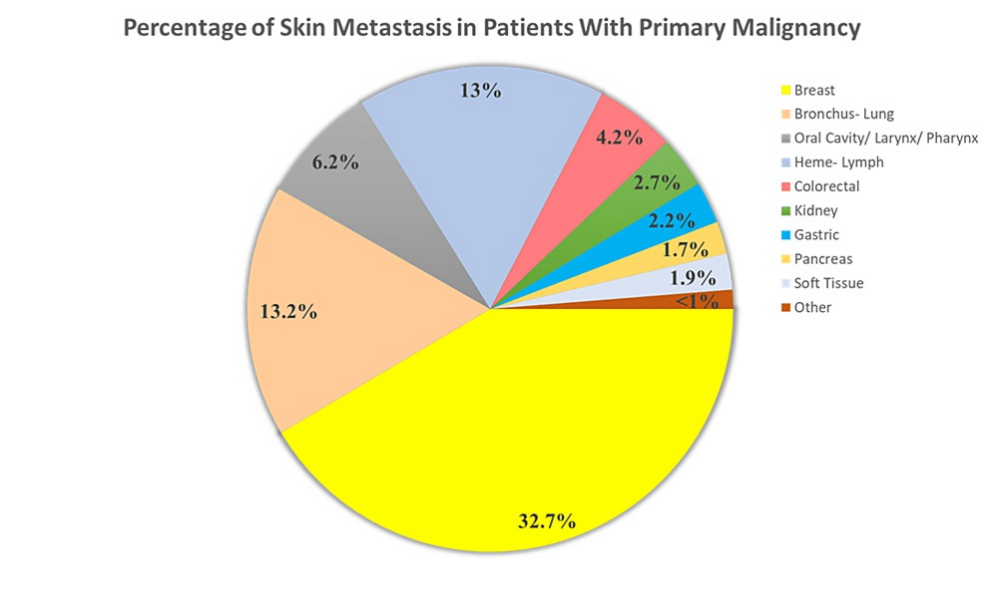
Percentage of skin metastasis in patients with primary malignancy *Note: *Adapted From "Cutaneous Metastasis of Internal Tumors," by E.A. Choate, A. Nobori, and S. Worswick. (Cutaneous Metastasis of Internal Tumors - ScienceDirect). Copyright 2019 by Elsevier Inc.

Cutaneous metastasis of internal malignancies is generally rare and encountered in 0.7-0.9% of patients with cancer. It may be the first manifestation of internal malignancy or portend cancer recurrence well after treatment of a primary tumor [5]. The presence of skin metastasis reflects widespread disease and denotes poor prognosis, with survival after diagnosis ranging from 1 to 34 months [3]. Figure 3 conveys the percentage of skin metastasis in patients with primary malignancy [6].

Among men and women, patterns of skin metastasis differ, with lung cancer, melanoma, and CRC being the most common etiology in men. In women, breast cancer, CRC, and melanoma are the frequent culprits [3]. The rate of cutaneous metastases originating from CRC is about 4.2% [1], with the most common location being the abdominal wall, affected as a result of direct extension to the overlying skin or by hematogenous or lymphatic dissemination [7]. The lesions usually occur close to the vicinity of the primary tumor, and although less frequent, distant metastasis of CRC most commonly occurs in the face and scalp [1,8,9]. Metastatic CRC skin lesions localized to the upper extremities are an extremely unusual site [7], thus presenting a diagnostic challenge to clinicians.

Metastatic skin lesions can assume different morphological features, with most presenting as single or multiple painless violaceous to flesh-colored skin nodules [10]. In a large series describing 29 cases of cutaneous CRC metastasis, they found that 70% of cutaneous metastasis presented as single lesions compared with 30% presenting as multiple lesions. Strikingly, all facial and thoracic skin metastasis emerged as single lesions, which is not often initially speculated to be metastasis. Therefore, any development of new changes in the skin should prompt evaluation for metastasis in the appropriate clinical context [11]. The lesions often mimic epidermal cysts, lipomas, neurofibromas, lymphoma, alopecia, cicatricial plaques, and infectious etiologies. Inflammatory skin metastasis resembling cellulitis has been reported as well, most often caused by breast cancer [8,10]. Interestingly, Liao et al. reported a case of synchronous breast and cutaneous metastasis, six years following abdominoperineal resection of CRC, resembling skin lesions from a primary breast malignancy [12].

In keeping with previous observations, Chiang et al. reported a case of cutaneous metastasis resembling herpes zoster rash in a 49-year-old patient with a history of metastatic colon cancer. The mechanism is yet to be understood, however, it has been suggested that the Koebner phenomenon in places of trauma from prior zoster infection or surgery can play a role in implantation of tumor cells [13]. Of note, sessile acrochordon-type lesions are distinctive cutaneous presentations of metastatic CRC [6].

In order to assemble and outline the available information regarding the metastatic cutaneous lesions of CRC, we have conducted an extensive review of the literature for the past five years and have summarized the findings in Table 1.

**Table 1:** Cases of colorectal cancer with cutaneous metastasis *BRAF V600E mutation positive Abbreviations: N/A: Not available; C: Chemotherapy; R: Radiation, CR: Chemoradiation therapy; AC: Adjuvant chemotherapy; ACR: Adjuvant chemoradiation therapy; NCR: Neoadjuvant chemoradiation therapy; APR: Abdominoperineal resection

Author	Age, years	Gender	Histology	Stage	Treatment of colon cancer	Location cutaneous metastasis	Skin morphology	Treatment of cutaneous metastasis	Positive Immuno-histochemistry	Interval	Prognosis
Correia et al., 2021 [1]	65	male	adenocarcinoma	stage 4	N/A	back and forearm	two nodules	resection	CK20 and CDX2	synchronous	3 days
Liao et al., 2020 [3]	62	female	moderately differentiated adenocarcinoma*	stage 3	radical surgery, AC	chest, back, armpit	multiple nodules	C	CK20, CDX2, and SATB2	7 months	12 weeks
Liao et al., 2021 [12]	68	female	tubular adenocarcinoma	stage 2	APR+ C	bilateral breasts	pruritic lump	C	CK20, CDX2, villin, CEA	7 years	3 months
Faenza et al., 2019 [14]	92	female	moderately differentiated adenocarcinoma	N/A	N/A	left supraclavicular area	single nodule	excision	CK AE1-AE3, CDX	N/A	1 month
Ahmad et al., 2019 [15]	51	male	moderately differentiated adenocarcinoma	N/A	C	penile shaft, scrotum, glans of the penis, perinium	multiple subcutaneous nodules	N/A	CK20, CDX-2	synchronous	N/A
Malla et al., 2019 [16]	35	male	mucinous adenocarcinoma	N/A	APR	face, chest, abdomen, back	multiple nodules	N/A	N/A	2 months	3 months
Qiang et al., 2019 [17]	55	female	adenocarcinoma	N/A	CR	vulva	nodules	CR	N/A	1 year	10 months
Samanci et al., 2020 [18]	45	male	adenocarcinoma	N/A	C	scalp, mandibular	subcutaneous lump	N/A	CDX-2, CK-20	5 months	3 months
Junak et al., 2020 [19]	83	female	poorly differentiated adenocarcinoma with squamous differentiation	stage 4	left colectomy	right midback	raised mass	WLE, C	CK20	6 weeks	N/A
Badiani et al., 2020 [20]	70	male	moderately differentiated adenocarcinoma	N/A	NCR, APR+ fistula tract resection	left perianal region	subcutaneous mass	N/A	N/A	synchronous	N/A
Ye et al., 2020 [21]	49	female	moderately differentiated adenocarcinoma	N/A	CR	left breast	several nodules	CR	CA153, villin, CDX2, and P53	18 years	3 months
Mandzhieva et al., 2020 [22]	66	male	signet ring cell adenocarcinoma	stage 3	proctectomy, CR	bilateral groin	vesicular papules	R	N/A	3 months	N/A
Hakami et al., 2020 [23]	45	male	moderately differentiated adenocarcinoma	stage 3	diversion loop colostomy	right inguinal area	multiple exophytic masses	N/A	CDX2	synchronous	N/A
Zhou et al., 2021 [24]	53	female	adenocarcinoma*	stage 4	C	left groin	nodule	C	SATB2, CK20, MLH1, MSH2, MSH6, PMS2, Ki67(+ 80%)	9 months	N/A
Faye et al., 2021 [25]	88	female	mucinous and low-grade Lieberkühn adenocarcinoma	stage 2	Right hemi-colectomy and ileo-transverse anastomosis	forehead	pseudo cystic lesion	resection	CDX2 and cytokeratin	11 months	N/A
De Giorgi et al., 2021 [26]	63	male	squamous cell carcinoma of the rectum	stage 3	N/A	left scrotum	numerous erythematous nodules	CR	AE1/AE3, CK5/6, p40, p63, CK7	9 months	3 months
Fong et al., 2021 [27]	70	female	invasive adenocarcinoma*	stage 3	right hemi-colectomy with ileo-colic anastomosis	upper right chest wall	multiloculated mass	C	N/A	synchronous	N/A
Alhuzimi et al., 2021 [28]	60	female	adenocarcinoma	N/A	C	periumbilical	plaque	N/A	CK20	2 months	1 month
Majdoubi et al., 2021 [29]	48	male	well-differentiated and infiltrating adenocarcinoma	stage 4	resection, AC	umbilicus	nodule	resection	N/A	synchronous	N/A

The majority of metastatic skin lesions are adenocarcinomas, and those originating from the large intestine are usually well-differentiated mucin-secreting carcinomas with a nodular appearance. The diagnosis is primarily made by histology of the skin lesions and screening immunohistochemical studies, which are particularly helpful in poorly differentiated or anaplastic tumors. Morphologic features may aid in the diagnosis as well [3,5].

It has been reported that the presence of a *BRAF* mutation is associated with a poorer prognosis in patients with CRC. Moreover, CRC associated with wild-type *BRAF* mutation varies in the clinicopathological aspects from mutated BRAF colon malignancy, regardless of the microsatellite instability status, which is important to consider for management purposes [3].

The management and treatment of cutaneous metastasis lacks a standardized policy and yet remains to be further explored. Chemotherapy and anti-vascular endothelial growth factor (VEGF) therapy are less successful in treating patients with *BRAF*-positive mutations [3]. Compared to standard chemotherapy, the updated results of the BEACON CRC study determined that encorafenib plus cetuximab - with or without binimetinib - improved overall survival in patients with *BRAF* V600E mutant metastatic CRC. This combination could be successfully implemented as the new standard of care for metastatic *BRAF* V600E mutant CRC, including subcutaneous metastases [30]. 

In the presented case, multiple rounds of chemotherapy failed to adequately control the disease, as evidenced by the progression of the skin metastasis. Ultimately, she was switched to palliative radiation in attempt to improve functionality and quality of life. 

Current advances in management of cutaneous lesions recommend surgical excision when feasible with the goal of reducing the tumor burden and improving quality of life. Many times, treatment of the primary tumor will diminish the cutaneous lesions [5]. Wide local excision of the metastatic colorectal skin lesions was recommended by Nesseries et al. for isolated cases, while palliative resection was advised for cases with extensive cutaneous metastasis [10]. Excision with restricted margins can be used for cosmetically sensitive areas or resection performed for palliative purposes. For unresectable lesions, palliative radiotherapy and systemic chemotherapy are possible options, although no consensus exists outlining the ideal chemotherapy regimen thus far [4].

The American Society of Clinical Oncology (ASCO) endorses physical examination to look out for cutaneous metastases in confirmed cases of CRC, as early diagnosis is fundamental for adequate management [31]. For this reason, we recommend expedient patient education for the timely detection of any newly developed cutaneous metastatic deposits.

## Conclusions

In conclusion, cutaneous metastasis of colorectal cancer is a rare but alarming phenomenon, often mimicking other pathologies, hence leading to misdiagnosis. It frequently signifies widespread visceral disease and portends a grave prognosis. Early detection with watchful physical examination is essential for appropriate and timely management, thus making biopsy followed by histological and immunohistochemical examination mandatory for newly developed cutaneous lesions in the appropriate clinical context. Current advances in management lack a standardized approach and remain yet to be further explored. Wide local excision is an option for isolated cases, while palliative chemotherapy or radiotherapy is offered for unresectable lesions to improve functionality and quality of life.
